# Sensorineural hearing loss in the acute phase of a single episode of acute otitis media^☆^

**DOI:** 10.1016/j.bjorl.2019.06.001

**Published:** 2019-07-02

**Authors:** Ana Luiza Papi Kasemodel, Ludmilla Emília Martins Costa, Rafael da Costa Monsanto, Andreza Tomaz, Norma de Oliveira Penido

**Affiliations:** Universidade Federal de São Paulo (UNIFESP), Escola Paulista de Medicina (EPM), Departamento de Otorrinolaringologia, Cirurgia de Cabeça e Pescoço, São Paulo, SP, Brazil

**Keywords:** Otitis media, Hearing loss, Sensorineural hearing loss, Otite média, Perda auditiva, Perda auditiva neurossensorial

## Abstract

**Introduction:**

Acute otitis media is a disease with high global prevalence, that can lead to several acute complications and auditory sequelae. Data regarding the auditory evaluation in the acute phase of acute otitis media are scarce.

**Objective:**

To evaluate the main audiometric changes (air and bone conduction thresholds) in the initial phase of an acute otitis media episode.

**Methods:**

A case-control study was performed. Patients diagnosed with acute otitis media with less than 7 days of evolution in relation to the complaint onset were selected, and healthy volunteers were selected as controls. The acute otitis media and control groups were submitted to pure tone and vocal audiometry.

**Results:**

The acute otitis media group included a total of 27 patients (30 ears). Hearing loss was present in 90.0% of the ears with acute otitis media, with conductive loss in 14 (46.67%) and mixed loss in 13 (43.33%). Both the air and bone conduction thresholds obtained with the tonal audiometry in the acute otitis media group were significantly worse than the controls at all tested frequencies (*p* < 0.05). In patients with acute otitis media, we observed that the thresholds for frequency >1 kHz (bone conduction) and 3 kHz (air conduction) were significantly worse in patients with tinnitus compared to patients without tinnitus.

**Conclusion:**

During the first 7 days of evolution after the onset of an isolated episode of acute otitis media, we observed significant increases in bone and air thresholds at all frequencies, especially >2 kHz, compared to healthy ears.

## Resumo

**Introdução:**

A otite média aguda é uma doença de elevada incidência global, que pode levar a diversas complicações agudas e sequelas auditivas. Dados referentes à avaliação auditiva na fase aguda da otite média aguda são escassos.

**Objetivo:**

Avaliar as principais alterações audiométricas (limiares em via aérea e óssea) na fase inicial de um episódio de otite média aguda.

**Método:**

Realizou-se estudo de caso-controle. Selecionamos pacientes com diagnóstico de otite média aguda, com menos de sete dias de evolução em relação ao início das queixas, e voluntários saudáveis foram selecionados como controles. Os grupos otite média aguda e controle foram submetidos a audiometria tonal, vocal e audiometria.

**Resultados:**

O grupo otite média aguda incluiu 27 pacientes (30 orelhas). Observou-se presença de perda auditiva em 90% das orelhas com otite média aguda, condutiva em 14 (46,67%) e mista em 13 (43,33%). Tanto os limiares auditivos por via aérea quanto os limiares por via óssea obtidos com audiometria tonal do grupo otite média aguda eram significativamente piores em relação aos controles, em todas as frequências testadas (*p* < 0,05). Em pacientes com otite média aguda, observamos que os limiares das frequências acima de 1 kHz (via óssea) e 3 kHz (via aérea) eram significantemente piores entre pacientes com zumbido em comparação a pacientes sem zumbido.

**Conclusão:**

Nos primeiros sete dias de evolução do quadro inicial de um episódio isolado de otite média aguda, observamos aumentos significativos dos limiares ósseos e aéreos em todas as frequências, principalmente nas acima de 2 kHz, em comparação a orelhas sadias.

## Introduction

Acute otitis media (AOM) is a disease with high global prevalence (10.85%), affecting approximately 709 million people/year.^1^ Otitis media is also associated with severe and potentially life-threatening acute complications, including intratemporal complications (acute mastoiditis and petrous apicitis, for example) and intracranial complications (such as sigmoid sinus thrombosis, intracranial abscess and meningitis).^2^, ^3^ Even in uncomplicated cases, several studies have shown that otitis media may lead to permanent sequelae, including hearing loss, tinnitus, and vestibular symptoms.^4^, ^5^, ^6^, ^7^, ^8^ Regarding the hearing loss, it is estimated that approximately 31 in every 10,000 people with different types of otitis media have impairing hearing loss (auditory thresholds >25 dBHL).^1^

It is known that AOM leads to conductive hearing loss in the acute phase of the disease. However, recent studies have also demonstrated varying degrees of sensorineural hearing loss, especially at high frequencies, (2–8 kHz) in patients with a history of acute or recurrent AOM.^4^, ^9^ Although the clinical impact of hearing loss at high frequencies has not been extensively studied in the past, it has been associated with deterioration of musical tone perception, difficulties in sound localization and speech perception (especially in noisy environments), and tinnitus.^10^, ^11^, ^12^, ^13^ Additionally, a 30-year cohort also showed that adults with a history of recurrent childhood AOM had worse auditory thresholds^14^ and a significantly higher prevalence of tinnitus,^13^, ^15^ as compared to individuals with no history of AOM.

Considering that the high frequencies are the most affected in AOM,^5^ auditory evaluation in the acute phase of the infectious process can be an important and early part of the identification of possible hearing sequela and acute complications, such as serous or suppurative labyrinthitis.^4^, ^16^, ^17^, ^18^ Thus, it is possible to early initiate appropriate treatment and increase the chances of hearing recovery. However, data regarding the evaluation of auditory alterations in the acute phase of AOM are scarce. Therefore, the aim of this study is to evaluate whether, in the acute phase, AOM leads to changes in auditory thresholds (bone and air conduction) as compared with healthy ears.

## Methods

This was a controlled, cross-sectional, clinical study, carried out from February to December 2018, using a non-probabilistic convenience sample. We also carried out the sample calculation, based on the availability of cases from our emergency service that fully met all the defined exclusion criteria. Assuming a sampling error of 15% and a 95% confidence level, the sample calculation resulted in 25 individuals.

Patients with a diagnosis of AOM were selected from the emergency room at a tertiary university hospital. In this study, AOM was clinically defined according to the following diagnostic criteria: (1) Acute onset of clinical signs and symptoms; (2) presence of middle ear effusion, indicated by the presence of bulging of the tympanic membrane, limited mobility of the tympanic membrane, air-fluid behind the tympanic membrane, and/or otorrhea; and (3) signs and symptoms of middle-ear inflammation, as indicated by either distinct erythema of the tympanic membrane or otalgia.^19^ Our study protocol was approved by the Research Ethics Committee of our institution (protocol number 0364/2017), and also followed the principles dictated by the declaration of Helsinki for studies in humans.^20^

The inclusion criteria were defined as patients older than 18 years, with less than 7 days between symptom onset and diagnosis. We excluded patients who (1) had previous otologic disease; (2) had hearing loss identified prior to acute otitis media; (3) had comorbidities that could lead to sensorineural hearing loss, such as metabolic, vascular and autoimmune diseases; (4) had clinical syndromes or external ear malformations; (5) had cognitive deficits (6) patients with a history of cranio-encephalic trauma; (7) with tympanic membrane perforation unrelated to the current episode of AOM (8) underwent previous otologic surgery; (8) had a history of chronic exposure to noise; and (9) had a history of cancer affecting the temporal bone, or subjected to systemic chemotherapy or radiotherapy in the head and neck region. The selected patients underwent conventional tonal (250 Hz–8 kHz) and speech audiometry; when the TM was intact, we also performed immittance testing. We analyzed bone conduction at the frequencies of 500 –4 kHz and the air conduction at frequencies of 250 Hz–8 kHz. We defined “hearing loss” as the presence of thresholds >25 dBHL at any tested frequency. The value of the difference between the thresholds obtained by bone and air conduction at each frequency was termed the “air-bone gap”. In cases where the interaural difference was ≥40 dB in the air or bone conduction, we used masking in the untested ear, since the attenuation is practically nil. The masking was performed using the plateau technique, developed by Hood,^21^ considering its effectiveness in cases where overmasking is possible. To reduce the risk of calibration bias, all audiometric measurements were performed by the same speech therapist, specialist in clinical audiology, using the same equipment.

Speech audiometry was analyzed using 2 parameters: (1) Speech Recognition Threshold (SRT); and (2) Speech Recognition Percentage Index (SRPI).^22^ The classification of the immittance testing was based on the criteria proposed by Jerger, according to the middle ear pressure, as type A (peak at +200 to −99 daPa), type C (peak at −100 to −400 daPa) or B (flat curve).^23^

For comparison with the results obtained with the study group (AOM), healthy individuals, with no previous history of otitis media were selected as controls. The controls were selected using the same inclusion and exclusion criteria applied to the AOM group, and underwent the same audiometric evaluation routine.

The nonparametric Mann–Whitney test was used to compare the bone and air thresholds of AOM patients with control individuals. The Spearman’s test was performed to correlate the variables age and bone auditory thresholds. The results of the statistical tests were considered significant when the value of *p* < 0.05.

## Results

### Demographic, anamnesis and physical examination data

The final group of patients with AOM consisted of 27 patients. Of these, 15 were males (55.5%) and 12 were females (44.5%), with a mean age of 36.13 years (median = 37.5 years, range = 18–67 years; Standard deviation [SD] = 11.77 years). In total, 30 ears with AOM were evaluated, since 3 patients (11.1%) had AOM in both ears. The control group consisted of 16 individuals (32 ears). In the control group, the mean age was 35.12 years (median = 35 years, range = 18–62 years, SD = 12.19 years) ; 8 were men (50%) and 8 were women (50%). There were no significant differences between the mean age and gender distribution between the AOM and Control Groups (*p* > 0.05).

The median time of evolution since the onset of AOM complaints to the clinical evaluation was 4.7 days. Regarding the clinical symptoms, 100% of the patients had otalgia; 81.5% had hearing loss; 81.5% had tinnitus; and 74.1% had aural fullness. All analyzed patients had at least two of the abovementioned clinical symptoms. Otoscopy of all patients with AOM disclosed the presence of hyperemia, and in 4 patients with AOM the presence of tympanic membrane perforation was observed. No individual in the control group had auditory complaints or alterations at the otoscopy.

### Audiometric results

**Figure 1:**
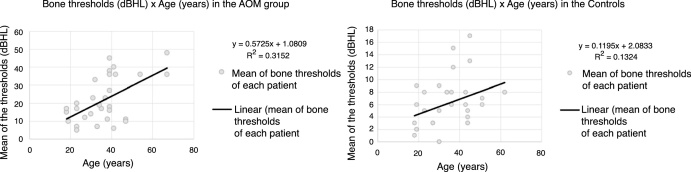
Scatter plot showing a positive correlation between age and sensorineural hearing loss in the ears with AOM and in controls.

Initially, we assessed the bone and air thresholds of the contralateral ear in patients with unilateral AOM compared to the audiometric measurements of the control group. This comparison did not show significant differences between any of the frequencies (*p* > 0.05). Of the 30 ears with AOM, it was observed that 27 (90%) showed hearing loss identified at the audiometry, with conductive loss in 14 (46.67%) and mixed loss in 13 (43.33%). In the 3 patients with normal audiometry, a comparative analysis was performed with the contralateral ear, which showed lower bone thresholds on the affected side in relation to the contralateral side, without air-bone gap (AOM, mean threshold = 15.0 dBHL, contralateral ear, mean = 5.33 dBHL, *p* > 0.05). In the control group, all subjects had audiometric results within normal values ​​(<25 dBHL). Both the control and the AOM groups showed a positive correlation between age and increase in bone thresholds (R = 0.509 and 0.457, respectively) (Fig. 1). In the control group, the tested bone thresholds were exactly the same as those obtained through air-conducted testing at all frequencies.

**Figure 2:**
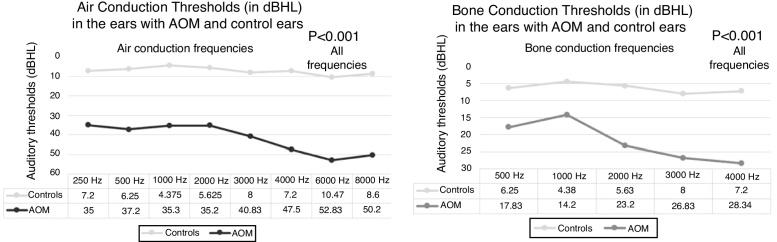
Line plot comparing the audiometric thresholds (air and bone) in the ears with AOM and control ears.

Comparative analysis of the audiometric results obtained by air conduction showed that-among patients with AOM - auditory thresholds were significantly worse compared to the control group (*p* < 0.001) (Fig. 2). To reduce the influence of alterations in sound conduction in patients with AOM, we also comparatively analyzed the results obtained with the bone conduction test in patients with AOM with the bone thresholds of the controls. This analysis also revealed that the bone thresholds of patients with AOM were also significantly worse than those of controls at all frequencies (*p* < 0.001) (Fig. 2).

**Figure 3:**
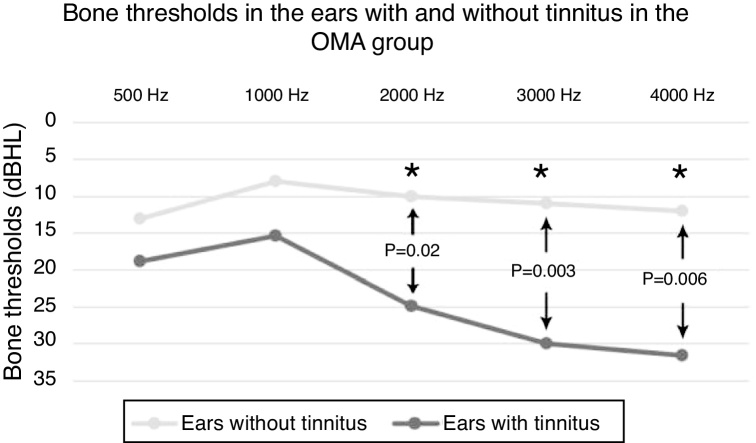
Line plot comparing the bone thresholds of the ears with and without tinnitus in the AOM group (acute otitis media). * Statistically significant differences (*p* < 0.05).

We also performed, in patients with AOM, a comparative evaluation between the bone and air conduction thresholds of patients with and without tinnitus. We found mean air conduction thresholds of 44 dBHL in the ears with tinnitus vs. 25.75 dBHL in the ears without tinnitus (Table 1). The mean bone thresholds of the ears with tinnitus were 23.96 dBHL vs. 10.8 dBHL of the ears without tinnitus (Fig. 3). The difference between the thresholds of ears with tinnitus and those without tinnitus was significant at frequencies from 2 kHz for the bone conduction and from 4 kHz for air conduction (Table 1).

**Table 1:** *p*-Values demonstrating (or not) significance between air conduction (AC) and bone conduction (BC) thresholds, when comparing the ears with and without tinnitus in the AOM group.

	250 Hz	500 Hz	1 kHz	2 kHz	3 kHz	4 kHz	6 kHz	8 kHz
BC		*p* = 0.482	*p* = 0.194	*p* = 0.02*	*p* = 0.003*	*p* = 0.006*		
AC	*p* = 0,07	*p* = 0.087	*p* = 0.109	*p* = 0.082	*p* = 0.082	*p* = 0.005*	*p* = 0.01*	*p* = 0.03*

*p-Value considered statistically significant (<0.05).

**Figure 4:**
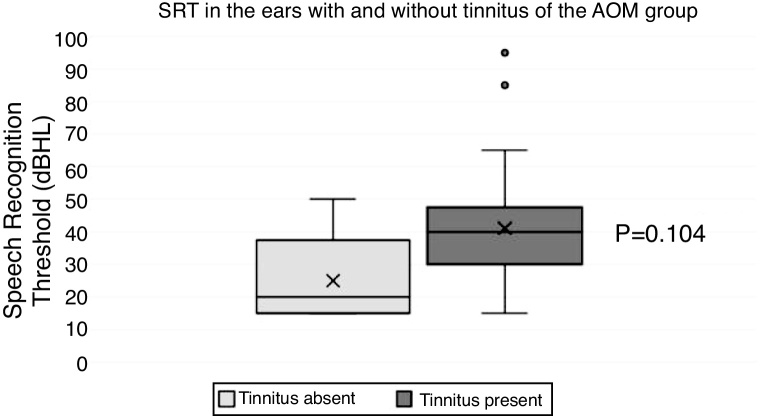
Boxplot chart showing the results of SRT in the ears with and without tinnitus in the AOM group.

Regarding the vocal audiometry, we observed worse values ​​of SRT (Speech Recognition Threshold) in the ears with AOM as compared with controls (*p* < 0.001) (mean SRT of 38.5 dBHL in the ears with AOM, mean SRT of 7.65 dBHL in the control ears). We observed a statistically significant difference when comparing the values ​​of SRPI (Speech Recognition Percentage Index) in the AOM group ears with the Control Group ears (*p* = 0.004). However, only two ears (6.67%) in the AOM group had SRPI values ​​below 92%. In the control group, 97% of the ears had SRPI of 100%, and only one ear in this group (3.1%) had an SRPI of 96%. Among patients with AOM, we observed worse SRT values ​​in the ears with tinnitus (mean SRT = 40.56 dBHL) when compared to ears without tinnitus (mean SRT = 27.5 dBHL); however, this difference was not statistically significant (*p* = 0.104) (Fig. 4). We also did not observe any significant differences between the SRPI of ears with and without tinnitus in the OMA Group (*p* = 0.313).

Regarding the immittance testing, 20 of 30 ears with AOM (66.67%) showed a type B curve, whereas 5 (16.67%) showed a type C curve. One ear showed a type A curve, and in 4 ears it was not possible to perform the immittance testing due to the presence of tympanic perforation.

## Discussion

Although AOM is a disease with a high incidence and prevalence that can result in permanent auditory sequelae, hypoacusis and the resulting hearing complaints are often undervalued.^24^ However, previous studies have demonstrated that the AOM can result in increased auditory thresholds, especially at high frequencies (8–16 kHz).^4^, ^9^, ^25^ However, the audiometric evaluation of patients with uncomplicated AOM is not routinely carried out, and it is performed only in more severe and complicated cases. Therefore, the lack of a routine auditory testing may contribute to the underdiagnosis of these sequelae.^26^

Among the 30 assessed ears with AOM, auditory threshold alterations were observed in 90%. Despite the observation that conductive deficits were slightly predominant (46.67%), the presence of mixed hearing loss affected 43.33% of the sample, suggesting that the presence of a sensorineural component may occur in a considerable number of patients with AOM. The prevalence found in this study was higher than that previously reported in the literature, ranging between 2.4%^9^ and 10.6%.^4^ A possible explanation for such differences is that this study was carried out at a tertiary care hospital, which usually receives patients referred by other institutions in cases of more severe diseases or with an atypical presentation.

The literature shows possible pathophysiological explanations for the presence of each type of hearing loss secondary to AOM: conductive deficits in the acute phase are mainly caused by the presence of fluid in the middle ear,^9^ and the sensorineural component probably occurs due to intracochlear inflammation generated by the passage of toxins and inflammatory agents from the middle ear to the inner ear through the round window membrane.^27^, ^28^, ^29^ This mechanism has been proven in animal models which have shown that the round window membrane is permeable to several inflammatory mediators.^27^, ^28^, ^29^ These mediators were mainly retrieved in the basal turn of the cochlea, which corresponds tonotopically to the high frequencies.^30^, ^31^, ^32^ Clinically, translational studies have also positively correlated the region in which the main pathological alterations were found (basal turn of the cochlea) to the auditory frequencies most frequently affected by the AOM (high frequencies).^28^ Another possible phenomenon that may lead to hearing loss at the early stage of AOM is the presence of transient endolymphatic hydrops, caused by pressure variations in the middle ear, round window membrane mobility alterations, or by the intracochlear inflammatory process. This phenomenon has already been demonstrated in clinical studies and also in temporal bones — Paparella et al. observed that more than 60% of 194 temporal bones with otitis media had some characteristic sign of hydrops.^33^

To minimize the impact of auditory conduction alterations on auditory thresholds analysis, we comparatively analyzed the bone-conducted audiometry thresholds in patients with AOM with the bone thresholds of controls. In our sample, we observed worse bone conduction thresholds at all frequencies (500 Hz–4 kHz) in the ears with AOM; this difference was more marked at the frequencies of 3 kHz and 4 kHz. In this sense, this study seems to be the first to perform this type of analysis during the acute phase of AOM in a group of patients. The case report published by Margolis and Nelson^34^ corroborate our findings: in this study, the authors observed that the sensorineural component preceded the onset of conduction alterations and was probably the result of the local inflammatory process that extended to the basal gyrus of the cochlea — this would occur even before the significant accumulation of fluid in the middle ear.^25^, ^34^ In our study, we observed a similar phenomenon in some ears: in three ears with AOM without air-bone gap and thresholds <25 dBHL, the thresholds were significantly worse as compared with the contralateral ear, suggesting possible acute functional injury. Additionally, the authors also observed that the affected frequencies were mainly above 2 kHz,^34^ another finding also observed in our study.

We observed that a significant proportion (81.5%) of patients with AOM complained of tinnitus. In these patients with tinnitus, auditory thresholds through bone conduction were significantly worse at frequencies >1 kHz compared to patients with AOM without tinnitus. Although the correlation of tinnitus with hearing loss at high frequencies has already been established,^35^ few studies have correlated tinnitus as a possible sequel of AOM.^9^ Cordeiro et al.^9^ demonstrated that the presence of tinnitus 6 months after the acute AOM episode was positively associated with the presence of worse thresholds the extended high-frequencies (>8 kHz) compared to patients without tinnitus. A possible explanation for these findings, from a pathophysiologic standpoint, is the association between otitis media and injury to the sensory epithelium of the cochlea, including a significant decrease in the population of inner and outer hair cells.^6^, ^36^ In this sense, there is preliminary evidence in the literature correlating these sensory lesions (mainly inner hair cell dysfunction and synaptopathy in the neural connection with inner hair cells)^35^, ^37^ with the presence of tinnitus. Therefore, it is plausible to state that more severe degrees of internal ear injury secondary to AOM also correlate with the increased occurrence of tinnitus.^35^

This study has some limitations that should be highlighted. As it was a cross-sectional study, we could not analyze whether the increase in bone thresholds was temporary or permanent, and we could not follow the evolution of patient complaints or otoscopic findings. The total number of patients included in this study, despite meeting the requirements defined in the sample calculation, was small. This fact probably derives from the strict and restrictive exclusion criteria applied, which – although having a negative impact on the number of possible cases – were necessary to reliably assess the impact of the initial phase of AOM on audiometric outcomes. Another limitation of this study is that, although the results obtained at the audiometric test through bone conduction minimize the impact of conductive hearing loss on the actual thresholds, they do not necessarily represent cochlear function.^38^ Despite these limitations, our results have direct clinical relevance. The high frequency of patients with mixed hearing loss suggests that even in patients with uncomplicated AOM, there is a significant alteration in cochlear function during the acute phase. Similarly, even in ears that did not have a bone conduction reduction below 25 dBHL (suggesting purely conductive hearing loss), the bone conduction thresholds were worse than those found in healthy ears. It is expected that this study will encourage further prospective studies to assess the late clinical and audiometric impact of these alterations observed in the acute phase of AOM.

## Conclusion

During the first 7 days of evolution after the onset of an isolated episode of AOM, we observed significant increases in bone thresholds at all frequencies (mainly above 2 kHz) in comparison to the air conduction thresholds in healthy ears.

## Funding

R.C.M and A.L.P.K received a scholarship from the Coordenação de Aperfeiçoamento Pessoal de Nível Superior (CAPES) (Finance code: 001.).

## Conflicts of interest

The authors declare no conflicts of interest.
